# Vaccine Effects on Heterogeneity in Susceptibility and Implications for Population Health Management

**DOI:** 10.1128/mBio.00796-17

**Published:** 2017-11-21

**Authors:** Kate E. Langwig, Andrew R. Wargo, Darbi R. Jones, Jessie R. Viss, Barbara J. Rutan, Nicholas A. Egan, Pedro Sá-Guimarães, Min Sun Kim, Gael Kurath, M. Gabriela M. Gomes, Marc Lipsitch

**Affiliations:** a Center for Communicable Disease Dynamics, Department of Epidemiology, Harvard T. H. Chan School of Public Health, Boston, Massachusetts, USA; b Virginia Institute of Marine Science, College of William and Mary, Gloucester Point, Virginia, USA; c Instituto Gulbenkian de Ciência, Oeiras, Portugal; d US Geological Survey, Western Fisheries Research Center, Seattle, Washington, USA; e Graduate School of Integrated Bioindustry, Sejong University, Seoul, Republic of Korea; f Liverpool School of Tropical Medicine, Liverpool, United Kingdom; g CIBIO-InBIO, Centro de Investigação em Biodiversidade e Recursos Genéticos, Universidade do Porto, Porto, Portugal; Georgetown University; Yale School of Public Health

**Keywords:** all-or-nothing vaccines, heterogeneity, infectious disease dynamics, mode of vaccine action, partially protective vaccine

## Abstract

Heterogeneity in host susceptibility is a key determinant of infectious disease dynamics but is rarely accounted for in assessment of disease control measures. Understanding how susceptibility is distributed in populations, and how control measures change this distribution, is integral to predicting the course of epidemics with and without interventions. Using multiple experimental and modeling approaches, we show that rainbow trout have relatively homogeneous susceptibility to infection with infectious hematopoietic necrosis virus and that vaccination increases heterogeneity in susceptibility in a nearly all-or-nothing fashion. In a simple transmission model with an *R*_0_ of 2, the highly heterogeneous vaccine protection would cause a 35 percentage-point reduction in outbreak size over an intervention inducing homogenous protection at the same mean level. More broadly, these findings provide validation of methodology that can help to reduce biases in predictions of vaccine impact in natural settings and provide insight into how vaccination shapes population susceptibility.

## IMPORTANCE

Differences among individuals influence transmission and spread of infectious diseases as well as the effectiveness of control measures. Control measures, such as vaccines, may provide leaky protection, protecting all hosts to an identical degree, or all-or-nothing protection, protecting some hosts completely while leaving others completely unprotected. This distinction can have a dramatic influence on disease dynamics, yet this distribution of protection is frequently unaccounted for in epidemiological models and estimates of vaccine efficacy. Here, we apply new methodology to experimentally examine host heterogeneity in susceptibility and mode of vaccine action as distinct components influencing disease outcome. Through multiple experiments and new modeling approaches, we show that the distribution of vaccine effects can be robustly estimated. These results offer new experimental and inferential methodology that can improve predictions of vaccine effectiveness and have broad applicability to human, wildlife, and ecosystem health.

## INTRODUCTION

Host heterogeneity influences infection dynamics and thereby the ability to predict the impact of control measures (1, 2). While much attention has been given to host heterogeneity in exposure (e.g., contact rates) (3–7), considerably less attention has been paid to heterogeneity in susceptibility to infection given exposure (8–10). Heterogeneity in susceptibility is typically incorporated into model structure through increased compartmentalization (11). Individuals in different compartments are assumed to have different susceptibilities, while those in the same compartment are presumed identical (12). In nature, heterogeneity in susceptibility is continuous (8, 13–17), and factors influencing susceptibility may be unknown, making assessment of susceptibility to infection a significant challenge in prediction of infectious disease epidemics.

Tools from the field of quantitative microbial risk assessment can aid in estimation of heterogeneity in susceptibility. Dose-response relationships from this field posit that increases in pathogen challenge dose result in an increasing probability of host infection (18). Highly susceptible hosts are likely to become infected at low challenge doses, and as dose increases, more-resistant hosts become increasingly likely to become infected (8, 16). The shape of the relationship between challenge dose and probability of infection allows for estimation of the extent of host heterogeneity in susceptibility (19). A null hypothesis of complete homogeneity (no variation) in susceptibility implies that (i) the per-pathogen probability of establishing infection is the same for each host and (ii) the probability that each host becomes infected through challenge with a particular pathogen dose is independent in repeated challenges (18).

This null hypothesis makes two testable predictions. (i) The increase in the probability of infection with challenge dose will be steep, in accordance with the exponential decline in the Poisson probability of escaping infection as dose increases. Departures from this null hypothesis will result in a less-steep dose-response curve (18), in which the probability of infection increases more slowly with challenge dose. (ii) Over a single inoculation event with a fixed duration, if hosts could be challenged multiple times with the same pathogen without having their immune status modified by each exposure, the result of previous challenges should give no indication of what to expect in a subsequent challenge. Put another way, the success of one challenge in a host is uncorrelated with the success in a subsequent challenge when all hosts are equally susceptible. Therefore, after two challenges, the number of hosts infected zero, one, or two times should be geometrically distributed, with a single unknown parameter corresponding to the probability of infection in a single challenge (20). In a continuous challenge, the frequency of hosts infected over a longer challenge duration should be equivalent to that after challenging hosts with a higher dose for a shorter time period. For example, the fraction of hosts infected in a 1-h challenge at a high dose should be the same as the fraction of hosts infected in a 2-h challenge at half that dose (21).

Heterogeneity in susceptibility to infection, or departure from this null hypothesis, may be present among unvaccinated individuals, vaccinated individuals, or both. Heterogeneity in susceptibility is particularly important when considering vaccine efficacy, because the mode of vaccine action can influence disease dynamics (9) and pathogen evolution (22). Vaccines protecting hosts in a so-called “all-or-nothing” fashion will afford complete protection to some fraction of hosts while leaving other hosts completely unprotected, producing heterogeneous susceptibility in vaccinated hosts. The other extreme is a “leaky” vaccine, which partially protects each host to an equal degree (9). Of course, intermediates between the all-or-nothing and leaky extremes are also possible. Distinguishing the epidemiological mode of action of vaccination is important because an all-or-nothing vaccine has a greater impact in reducing population-wide pathogen transmission than a leaky vaccine of the same overall efficacy (9, 23–25). This is in part because under a given force of infection, the all-or-nothing vaccinated population will experience a susceptibility reduction over time due to cohort selection (more susceptible individuals are infected first and removed from the susceptibility pool). This effect is weaker when susceptibility is more homogeneous (8, 16, 26–28). Despite the importance of the leaky and all-or-nothing distinction, the incorporation of mode of vaccine action into epidemiological models is frequently determined by convenience rather than accuracy criteria.

Infectious hematopoietic necrosis virus (IHNV) is a single-stranded RNA virus endemic to salmonid fishes in the Pacific Northwest of North America (29, 30). The virus is a significant pathogen of wild, farmed, and hatchery salmonids, including rainbow trout (*Oncorhynchus mykiss*) (30, 31). The pathogen causes acute mortality due to necrosis of the host kidney and spleen (30). Due to the economic importance of rainbow trout, vaccines for IHNV have been developed that reduce mortality from disease and are cross-protective against multiple IHNV strains (32–39). In addition, selective breeding of fish for resistance to the virus has also been pursued (40–42). Multiple genotypes of the virus have also been found to circulate simultaneously (43, 44), and fitness differences among several viral strains have been previously characterized (45, 46). Several pathogen strains (genotypes B and C, described in detail below) have been shown to have nearly equivalent fitnesses with no evidence for strain interference effects (46). Because of the manipulability of rainbow trout, the ability to breed large numbers of pathogen-free individuals in captivity, knowledge of IHNV viral strain fitness, and the range of disease interventions against IHNV, this is an ideal model system for studying heterogeneity in innate and vaccine-induced susceptibility.

Here, we examine the distribution of host susceptibility to IHNV infection in unvaccinated and vaccinated fish using two experimental approaches motivated by the two predictions of the null model listed above. First, we investigate how increases in pathogen concentration influence infection in unvaccinated and vaccinated hosts, testing the first prediction of the null model of homogeneous susceptibility. Second, we test the second prediction of the null model by simultaneously challenging fish with two IHNV strains with similar fitnesses and compare single and dual infection frequencies to independently estimate susceptibility distributions and the mode of vaccine action. We use simultaneous challenge instead of repeated challenges to circumvent development of immunity following a primary exposure. Finally, we examine the impact of excluding vaccine-induced changes in heterogeneity in susceptibility on disease dynamics using a simple transmission model.

## RESULTS

**FIG 1:**
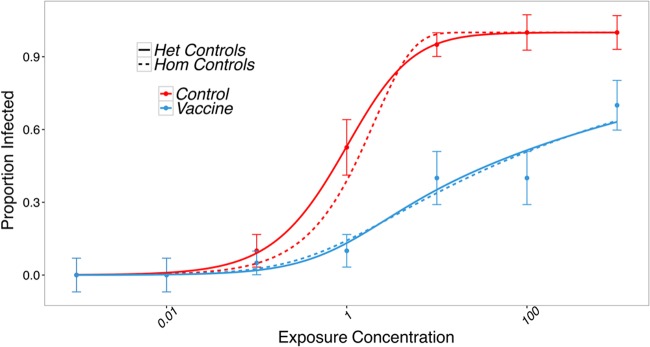
Best-fitting models (lines) for infection response of vaccinated and unvaccinated fish challenged with escalating concentrations of IHNV in PFU (plaque forming units) per microliter (points ± standard errors). Dashed lines show model fit where all unvaccinated individuals were equally susceptible (homogeneous) and vaccinated individuals had heterogeneous susceptibility distributed according to a beta distribution. Solid lines show model fit allowing for gamma-distributed heterogeneity in susceptibility in unvaccinated fish. Vaccinated fish have gamma-beta-distributed heterogeneity in susceptibility where parameters of the gamma distribution are determined by heterogeneity in susceptibility of the unvaccinated fish.

**FIG 2:**
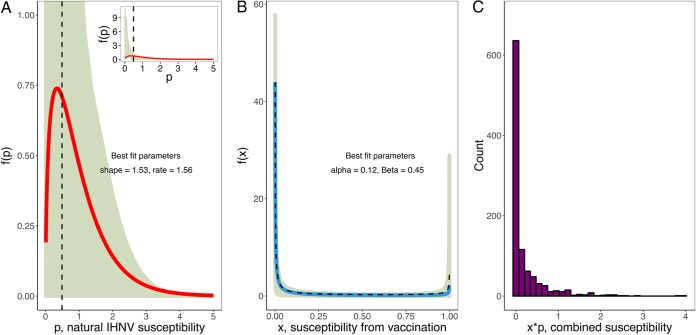
Estimated IHNV susceptibility distributions from models in Fig. 1. Solid lines show estimated distributions allowing heterogeneity in unvaccinated and vaccinated fish. Dashed lines show model fits allowing heterogeneity in the vaccine group only, with homogeneous controls. (A) Gamma-distributed susceptibility (microliters per PFU-hour) of unvaccinated fish challenged with IHNV. (Inset) Estimated gamma distribution showing full confidence interval range. (B) Beta-distributed susceptibility multiplier of vaccination, obtained under the assumption that a vaccinated fish’s susceptibility was the product of a random draw from the gamma distribution obtained from unvaccinated fish, multiplied by an independent random draw from this beta distribution. We obtained 95% confidence regions by bootstrapping chi-squared residuals to create 1,000 pseudoreplicates of infection data and then refitting the model to pseudoreplicates to determine the 95% confidence regions of parameters as described in reference 18. The dashed curve, almost indistinguishable from the solid blue one, is the pure beta distribution under the assumption of heterogeneity only in vaccinated hosts. (C) Histogram of the product of 1,000 random draws from a beta distribution multiplied by 1,000 random draws from a gamma distribution with parameters defined by distributions (colored lines) in panels B and C.

We first exposed groups of unvaccinated and vaccinated lab-reared juvenile rainbow trout to increasing concentrations of IHNV through immersion challenge. The probability of infection for unvaccinated fish increased steeply with exposure concentration (proportional to dose), consistent with relatively homogeneous susceptibility (Fig. 1), although a model with modest gamma-distributed heterogeneity in susceptibility in unvaccinated fish (mean = 0.979, variance = 0.656, shape = 1.53, rate = 1.56) (Fig. 2A) received slightly higher support (ΔBIC [Bayesian information criterion] = 2.47) (Fig. 1). The probability of infection at a given exposure concentration was consistently lower for vaccinated fish and increased more slowly with concentration, implying greater heterogeneity in susceptibility (18). There was no support for homogeneous susceptibility among vaccinated individuals (ΔBIC = 59.27) (see Table S1 in the supplemental material).

10.1128/mBio.00796-17.3 TABLE S1  Dose-response model comparisons of heterogeneous (beta and gamma) and homogeneous models. Download TABLE S1, DOCX file, 0.05 MB. Copyright © 2017 Langwig et al. 2017 Langwig et al. This content is distributed under the terms of the Creative Commons Attribution 4.0 International license.

Assuming that the distribution of vaccine effects in reducing susceptibility was gamma distributed and independent of baseline susceptibility, we obtained the beta distribution for vaccine action that produced the best-fitting combined beta-gamma distribution of susceptibilities for vaccinated hosts (Fig. 2B). The resulting beta distribution indicated a highly heterogeneous and polarized mode of vaccine action that conferred almost total protection on a majority of fish while a smaller fraction of individuals were nearly completely unprotected (α = 0.12, β = 0.45) (Fig. 2B). The estimated beta distribution assuming homogeneity of unvaccinated fish was nearly identical to the estimated beta distribution with gamma-distributed baseline susceptibility (Fig. 2B, black-dashed line). The resulting combined distribution assuming that the vaccine acts multiplicatively and independently on the innate susceptibilities of unvaccinated fish is unimodal with the mass of the distribution centered close to 0. This is due to the much larger mode of the estimated beta distribution of vaccine susceptibility multipliers near 0, as the majority of hosts were nearly completely protected by vaccination (Fig. 2C).

**FIG 3:**
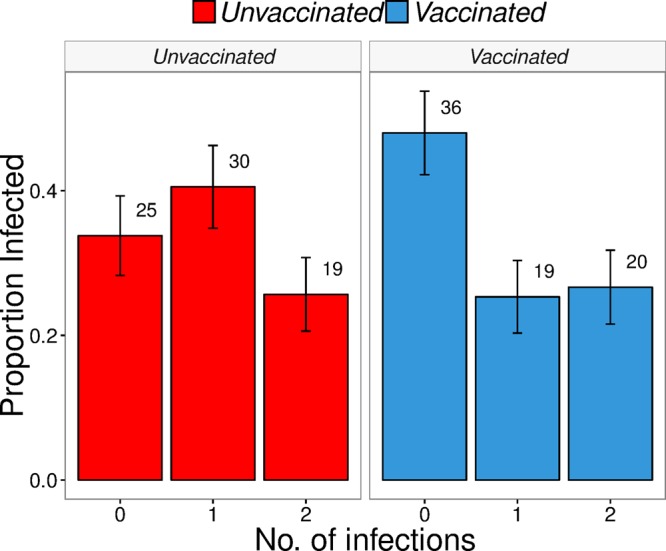
Fraction of vaccinated and unvaccinated fish with 0, 1, or 2 IHNV strains in a single-dose immersion challenge with 74 unvaccinated and 75 vaccinated fish. Numbers indicate the number of hosts infected in each group.

**FIG 4:**
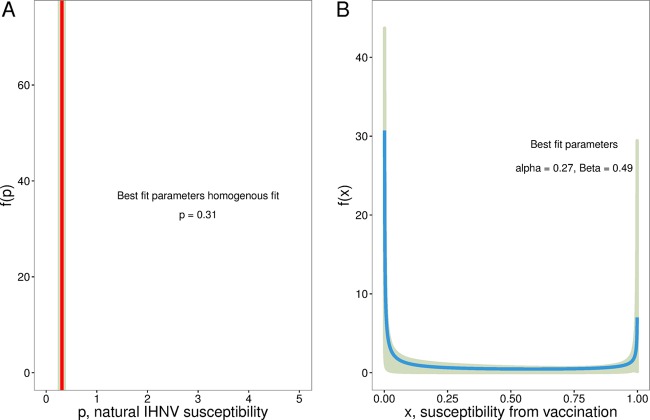
Estimated IHNV susceptibility distributions from simultaneous challenge of unvaccinated and vaccinated fish with two strains of IHNV. (A) Homogeneously distributed susceptibility (microliters per PFU-hour) of unvaccinated fish challenged with IHNV. (B) Beta-distributed susceptibility effect of vaccination. We obtained 95% confidence regions by bootstrapping observations to create 1,000 pseudoreplicates of infection data and then refitted the model to pseudoreplicates to determine the 95% confidence regions of parameters as described in reference 18.

To test the second prediction of the homogeneity model, we designed an experiment to determine whether hosts challenged twice with the same pathogen had independent probabilities of infection among challenges. If hosts were identical, the result of a single pathogen challenge should provide no information about the relative susceptibilities of individual hosts. To assess this, we challenged vaccinated and unvaccinated hosts with two viral strains with similar fitnesses at a single dose sufficient to infect approximately 50% of all vaccinated hosts in a single strain challenge. This was meant to simulate multiple sequential challenges (47) that could distinguish between leaky and all-or-nothing modes of vaccine action (10, 27, 48, 49), such that leaky protection would independently reduce susceptibility of a fish to each challenge while an all-or-nothing vaccine would protect a fish either completely against both challenges or not at all against either. Because challenge with IHNV induces a nonspecific immune response that makes a fish temporarily refractory to further infection (50), we used simultaneous challenges rather than sequential ones. Under a strictly leaky mode of action at a dose where 50% of vaccinated individuals become infected in a dose-response challenge, we would expect that 25% of vaccinated hosts would have no infections, 50% would have single infections, and 25% would have double infections, if the vaccine was exactly 50% effective against each strain. A strictly all-or-nothing mode of action would result in 50% of the hosts with no infections and 50% of hosts with double infections. We found that 48.0% of vaccinated hosts in the simultaneous challenge had no infections, 25.3% had single infections, and 26.7% had double infections (Fig. 3). In unvaccinated fish, 26% had no infections, 41% had single infections, and 34% had double infections. In unvaccinated fish, we found that the homogeneous model had slightly more support (ΔBIC = 0.498) (Fig. 4A; Table S2) than the heterogeneous model. This is consistent with the independent sorting of pathogen strains among identical individuals, although population probabilities were not perfectly leaky (e.g., 25% uninfected, 50% singly infected, and 25% double infected; chi-squared goodness-of-fit test, *P* = 0.15). Vaccinated fish were more heterogeneous (α = 0.27, β = 0.49, variance = 0.13) (Fig. 4B), and there was no support for homogeneous susceptibility (Table S2). Parameter estimates were within the 95% confidence interval of the distribution of vaccine effects estimated from the first experiment.

10.1128/mBio.00796-17.4 TABLE S2  Two-strain experiment model comparisons of heterogeneous (beta and gamma) and homogeneous models. Download TABLE S2, DOCX file, 0.05 MB. Copyright © 2017 Langwig et al. 2017 Langwig et al. This content is distributed under the terms of the Creative Commons Attribution 4.0 International license.

**FIG 5:**
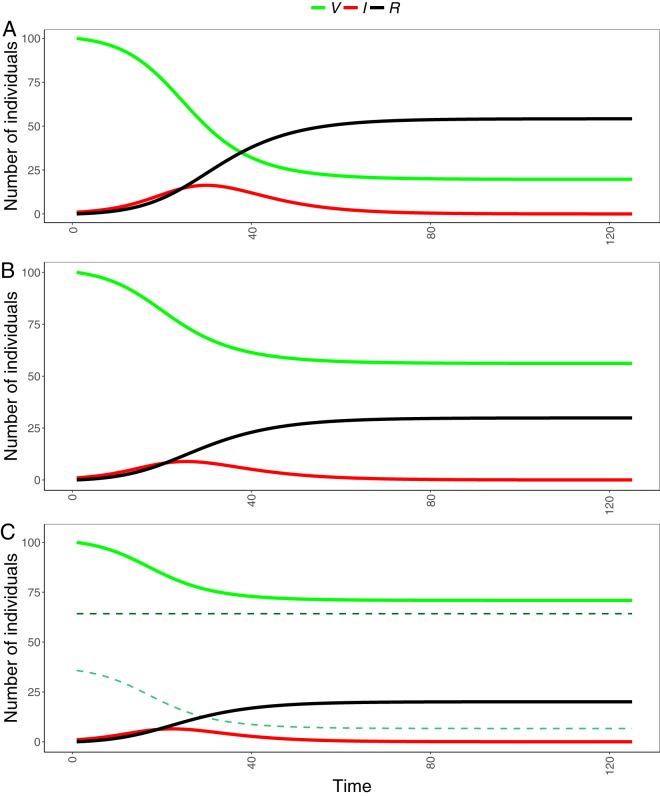
Disease dynamics of a susceptible-infected-recovered pathogen with disease-caused mortality, where *R*_0_ equals 2, without heterogeneity in susceptibility (A), with beta-distributed heterogeneity in susceptibility from experimental estimates (B), and with discrete all-or-nothing heterogeneity in susceptibility (C) (pale green dashed line, no vaccine protection; dark green dashed line, complete vaccine protection). The model is formally represented as the rates of change in a population of vaccinated (V), infected (I), and recovered (R) individuals. Susceptibility and vaccine protection parameters were determined by estimates of the beta distribution from the two-strain challenge experiment. Transmission, recovery, and disease-caused mortality parameters were not estimated from data. See Fig. S2 for the full range of *R*_0_ values explored in model simulations.

We used the estimated distribution of vaccine effects to examine the influence of experimentally estimated heterogeneity in susceptibility on disease dynamics, compared to models where the reduction in susceptibility due to vaccination was either homogeneous or discretely all-or-nothing (Fig. 5). At an *R*_0_ of 2, we found that in an epidemic of a vaccinated population, the experimentally estimated polarized distribution of vaccine effects reduced the total number of hosts infected by 35 percentage-points over a model where all individuals were assumed to be homogeneously protected to the same extent by vaccination (e.g., 45% of hosts became infected if vaccine protection was heterogeneous and 80% became infected if protection was homogeneous). However, this reduction was far less than the discrete all-or-nothing model, where only 36% of hosts became infected. The highly polarized mode of vaccine action caused the most susceptible individuals to become infected first and resulted in a reduction in mean susceptibility over the course of the epidemic (Fig. S1). At *R*_0_ values of >6, homogeneous vaccine protection resulted in 100% of individuals becoming infected (e.g., a final outbreak size of 100), whereas at an *R*_0_ of 25, heterogeneous protection, as estimated by our experiments, still prevented infection in 17% of vaccinated individuals (Fig. S2).

10.1128/mBio.00796-17.1 FIG S1  Number of individuals infected and change in susceptibility of the vaccine group from the simulated epidemic in Fig. 5B. (Top) The epidemic curve or the total number of individuals in the infected class from the heterogeneous model simulation. (Bottom) The change in susceptibility of the vaccinated group over the course of the simulated epidemic. The gray facet labels (1 to 120) correspond to the time points of the epidemic (top), and each panel is a snapshot of the susceptibility distribution at that time point. The *x* axis denotes the susceptibility value of each bin or class, and the *y* axis shows the number of individuals in each respective class. The mean value displayed on each panel shows the mean susceptibility of the vaccinated population at that time point. The mean decreases over the time course of the epidemic because individuals with the highest susceptibility become infected at earlier time points (e.g., 1 to 40), and individuals remaining in the vaccinated population are therefore relatively resistant. Download FIG S1, PDF file, 0.1 MB. Copyright © 2017 Langwig et al. 2017 Langwig et al. This content is distributed under the terms of the Creative Commons Attribution 4.0 International license.

10.1128/mBio.00796-17.2 FIG S2  Change in outbreak size with *R*_0_ from simulated vaccinated-infected-recovered disease dynamics without heterogeneity in susceptibility (homogeneous), with beta-distributed heterogeneity in susceptibility from experimental estimates (heterogeneous), and with discrete all-or-nothing heterogeneity in susceptibility (all-or-nothing). We varied *R*_0_ by varying only the transmission parameter (*v*), with δ = 0.05, and γ = 0.1 for all simulations. The dashed black line shows the value of *R*_0_ in the simulation shown in Fig. 5. The dashed gray line shows the *R*_0_ value where homogeneous susceptibility results in 100% of the vaccinated population (100/100 individuals) becoming infected over the entire epidemic. Download FIG S2, PDF file, 0.01 MB. Copyright © 2017 Langwig et al. 2017 Langwig et al. This content is distributed under the terms of the Creative Commons Attribution 4.0 International license.

## DISCUSSION

Heterogeneity in host susceptibility to infection is an often overlooked but important determinant of infectious disease dynamics (8, 9). In this study, we show that vaccination of rainbow trout increases heterogeneity in susceptibility to infection in a highly polarized fashion. Our findings of nearly identical susceptibility distributions using two independent methodologies affirm the utility of tools from quantitative microbial risk assessment in describing host heterogeneity and provide biological support for some polarized, nearly all-or-nothing modes of vaccine action, as found in this case. Furthermore, we provide evidence that a null model of homogeneous susceptibility does not adequately describe vaccinated host populations. However, unvaccinated hosts had more homogeneous susceptibility, with probability of infection increasing sharply with pathogen dose. We also found that two viral strains of equivalent fitnesses nearly independently sorted among unvaccinated hosts, confirming the null model of primarily homogeneous susceptibility in this group.

The robust estimation of susceptibility distributions using multiple methods provides several potential avenues for including heterogeneity in epidemic models and estimating vaccine effects across disease systems. Frequently, heterogeneity in susceptibility among hosts is unknown or is incorporated into model structure through numerous classes representing factors thought to be important determinants of susceptibility (e.g., age or sex) (51). Models may also incorporate heterogeneity in susceptibility based on risk factors identified in previous studies (e.g., tuberculosis infection in HIV susceptibility). However, even the most rigorously designed studies may overlook important underlying risks that influence host susceptibility. The tools presented here allow for assessment of all underlying variation in susceptibility and do not require understanding of individual risks or a mechanistic understanding of the causes of heterogeneity.

These results build on a large body of literature underscoring the importance of heterogeneity in infectious disease systems, including contact structure heterogeneity and variation in infectiousness (52, 53). Heterogeneity in infectiousness can modify disease dynamics, but importantly must be correlated with susceptibility, contacts, or other parameters (e.g., mortality from disease) to influence the size of epidemics. This is because the mean of the distribution will be identical among infected cohorts unless other model parameters preferentially remove or add individuals that change this mean (e.g., individuals that are more infectious die before they transmit). Conversely, like heterogeneity in susceptibility, heterogeneity in contacts will change dynamically over an epidemic as the individuals with the highest numbers of contacts become infected early in the epidemic and continue to infect others during their infectious period but then exit the infected pool as they recover (for pathogens with a recovered class). Therefore, adding heterogeneity in the encounter rate should further reduce the size of outbreaks, dependent on correlations between model parameters and the relative importance of each host trait in determining infection and mortality (54).

Rectifying diverse estimates of vaccine efficacy across studies is a significant challenge in public health (26). Our study further affirms that heterogeneous susceptibility among groups is a potential explanation for variation in these estimates. As expected, excluding heterogeneity in susceptibility from epidemic models had a significant impact on disease dynamics. Using a polarized mode of vaccine action resulted in fewer infected individuals under a wide range of *R*_0_ values, whereas homogeneous protection led to all hosts becoming infected for *R*_0_ values of >6. While the fraction of hosts infected will depend on particular parameter estimates, and we have not exhausted all parameter combinations and shape possibilities, this study provides further evidence that heterogeneous susceptibility decreases the size of epidemics. This is because the polarized mode of vaccine action greatly reduced the susceptibility of a fraction of individuals, nearly completely protecting them against infection. Transitions of susceptible individuals into the infected class occurred nonuniformly, with the most susceptible individuals first becoming infected, leaving increasingly resistant individuals in the susceptible class as the epidemic progressed. Conversely, the homogeneous model assumed that the susceptibility of all individuals was identical and therefore did not allow for the effect of cohort selection (55). The discrete all-or-nothing model overestimated vaccine protection compared to the experimentally derived distribution of vaccine protection. This further emphasizes that empirical estimates of vaccine protection can vastly improve epidemiological predictions and provide insight into vaccine programs (56).

In considering the design and interpretation of vaccine trials, it is important to understand the influence that variation in vaccine effects (9) and variation in natural susceptibility (57) will have on vaccine efficacy. In a vaccine trial, there will be a faster reduction in susceptibility in the control group relative to those individuals receiving vaccination, if the vaccine reduces susceptibility and prevents infection. This reduction in susceptibility will result in a reduction in incidence over time in the control group, and this effect can be misconstrued as waning vaccine efficacy (26). Therefore, it is important to consider that variation in susceptibility within and among both vaccinated and unvaccinated populations may contribute to perceived changes in vaccine efficacy over space and time, which highlights the necessity to consider alternative metrics for calculating vaccine efficacy (26, 58). Examination of vaccine study efficacy with epidemiological models can aid in reducing biases that may be overlooked using more static measures of vaccine efficacy (49).

Few studies have sought to investigate the mode of protection of control measures, as separating natural host heterogeneity from control-induced changes in susceptibility is a significant challenge (17). We provide a method for separating natural heterogeneity in susceptibility from changes in susceptibility due to vaccination, assuming that natural and vaccine-induced susceptibilities are independent. It is important to note that we did not explore all possible probability distributions, and the fit of different probability distributions is difficult to distinguish from our data (see Table S1 in the supplemental material). Future studies with larger sample sizes and greater dose ranges may be better able to assess the suitability of specific probability distributions, including the exploration of scaled distributions, or multiple distributions, which would allow for bimodal susceptibility without constraining the values to fall between 0 and 1. Future experiments should also be conducted to help determine whether natural and control measure susceptibility distributions should be treated independently and the influence that this may have on control measure efficacy.

Susceptibility of the unvaccinated hosts was very homogenous relative to those vaccinated. Rainbow trout lines used in this experiment have been selectively bred for disease resistance, among other traits, due to the importance of IHNV in disease outbreaks in aquaculture (42, 59, 60). It is possible that selective breeding may have contributed to decreased variation in susceptibility if hosts with lower susceptibility were selected from a population with greater variance. If selection is strongly directional and uncorrelated with other traits, susceptibility to a specific pathogen would be predicted to decrease and become more homogeneous over time, just as mean susceptibility decreases over the course of an epidemic (Fig. S1). Genetic variation is an important determinant of pathogen transmission and spread (61), and our results offer potential insight into the mechanism by which pathogens may spread more easily through homogeneous host populations. However, more research is needed to determine whether natural selection by pathogens might also select for relatively homogeneous susceptibility, given that hosts naturally exposed to pathogens likely experience more significant tradeoffs than hosts that have undergone purifying artificial selection (62, 63). Nonetheless, if selective breeding for disease resistance reduces variation in susceptibility, this will result in larger epidemics than if mean susceptibility is reduced by the same amount while preserving trait variance, as demonstrated in Fig. 5.

Heterogeneity in susceptibility is a key determinant of disease dynamics and impacts (8, 9, 14, 16, 17, 62). Although the importance of interindividual variation in susceptibility and risk response to disease is widely recognized (51), epidemiological models frequently fail to account for this variation. Our data emphasize the importance of incorporating heterogeneity in susceptibility in estimates of control measure efficacy. Inclusion of this variance will help to improve prediction and allow for better management of infectious diseases of humans and wildlife.

## MATERIALS AND METHODS

### Experimental methods.

Hosts used for all experiments were 1- to 3-g research-grade rainbow trout (*Oncorhynchus mykiss*) fry obtained from Clear Springs Foods Inc. Fish were maintained on specific-pathogen-free, sand-filtered, UV-irradiated water at 15 ± 2°C and fed 1.0-mm salmon feed (Skretting) daily at 1 to 2% per body weight. Viruses used in the experiments were IHNV genotypes HV, B, and C, previously referred to as 220:90, FF020-91, and FF030-91, respectively (46, 64). Genotype HV has previously been shown to have high *in vivo* fitness and virulence (45, 64, 65), and genotypes B and C have been shown to have equal *in vivo* fitnesses as measured by infectivity and in-host replication and virulence as measured by host mortality (46), in rainbow trout. Virus stocks were generated by culture on epithelioma papulosum cyprini (EPC) cells as previously described (38, 66), measured for infectious virus titer using plaque assays (46, 67), and stored at −80°C until experimental challenges. Prior to vaccination, fish were anesthetized briefly by immersion in 100 mg tricaine methanesulfonate buffered with 300 mg sodium bicarbonate in 1 liter H_2_O. Fish were vaccinated by intramuscular injection of 0.05 µg of the DNA vaccine pIHNwG, delivered in 25 µl of phosphate-buffered saline (PBS), as previously described (38). Sham-vaccinated fish received an injection of 25 µl of PBS. After vaccination, fish were allowed to fully recover before distribution into holding tanks. Fish were then held for 28 to 30 days prior to experimental exposure to virus to allow for immunity to fully develop (39) and weighed 1 to 3 g at the initiation of pathogen challenge experiments. Previous studies have shown that this vaccination regime is highly protective against IHN disease (38, 68, 69). The dose-response challenge experiment was conducted at the USGS Western Fisheries Research Center, Seattle, WA. The multiple-strain experiment was conducted at the Virginia Institute of Marine Science, Gloucester Point, VA.

For the dose-response experiment, we challenged subgroups of 20 pIHNwG-vaccinated and 20 sham-vaccinated fish with each of seven concentrations (0, 0.01, 0.1, 1, 10^1^, 10^2^, and 10^3^ PFU/μl) of IHNV genotype HV, creating a total of 14 treatment groups. Inocula were prepared in minimal essential medium with 10% fetal bovine serum (Gibco), such that virus was delivered as a 5-ml volume to each group. Fish were exposed to virus in batch, using a 1-h immersion in 1 liter of water containing virus, followed by a 1-h rinse in flowing water to remove exposure virus, as previously described (46). We then isolated fish into 1-liter beakers containing 400 ml of water and held fish under static conditions at 15°C for 3 days, the time period in which previous studies have demonstrated peak viral loads in infected fish (46, 70). Fish were then euthanized with 300 mg of tricaine methanesulfonate buffered with 900 mg of sodium bicarbonate in 1 liter of H_2_O and stored individually until RNA extraction as outlined below.

For the multiple-strain experiment, 75 vaccinated and 74 unvaccinated fish were simultaneously exposed to IHNV genotypes B and C at a concentration of 2 PFU/μl of each genotype, through an immersion challenge as described above except that the immersion volume was 4 liters and isolation tanks were 0.8 liter. On day 3 postexposure, fish were harvested and stored at −80°C as described above until viral load quantification.

Viral RNA was extracted from whole fish using the QIAamp Cador pathogen minikit (Qiagen) according to the manufacturer’s specifications, with the following modifications. Whole fish were homogenized by placing them in 4.5-ml Tallprep lysing matrix D tubes (MP Bio) with 1 ml guanidinium-thiocyanate solution (45) per gram of fish and then sonicated three times at 4.0 m/s for 20 s on a FastPrep 24 homogenizer (MP Bio) until completely liquefied. Homogenized fish were then centrifuged at 2,000 rpm and 20°C for 3 min, and 200 μl of the supernatant was combined with buffer VXL (Qiagen) and then run through the Cador kit protocol, with 100 μl buffer AVE used for elution. Extracted RNA was stored at −80°C until reverse transcription as previously described (45). Fish infection status was then determined by real-time quantitative PCR targeting the virus nucleoprotein (N) gene with primers IHNV N 796F and IHNV N 875R and TaqMan probe (Applied Biosystems) IHNV N 818MGB as previously described (71). All studies and protocols were approved under Virginia Institute of Marine Science IACUC-2013-02-11-8458-arwargo. This is in compliance with federal regulations (Department of Agriculture 9 CFR parts 1 and 2; Public Health Service 99-158).

### Inferential framework.

We modified dose-response models to describe the susceptibility of vaccinated and unvaccinated fish challenged with IHNV (18, 19, 72). We expanded the inferential framework from the conventional dose-response experiment to incorporate multiple viral strains. We fitted the models to data by either minimizing the deviance between the data and the model (dose-response experiment) (18) or minimizing the negative log likelihood using the multinomial distribution with classes zero-strain infections, single-strain infections, and two-strain infection (multiple-strain challenge). We jointly fitted the control and vaccine data by optimizing the parameters to minimize the sum of the deviance or of the negative log likelihood across both groups. We assumed that the baseline susceptibility in unvaccinated hosts followed a gamma distribution (19) and a beta distribution of vaccine effects to allow for bimodal (polarized) distributions (9, 17).

In equation 1, we allowed the unvaccinated control group to have homogeneous susceptibility, and susceptibility of the vaccinated group follows a beta distribution.

$$I_{C_{\text{hom}}}=1-e^{-pd}$$

(1) $$I_{V_{\text{het}}}=1-\int_{0}^{1}e^{-xpd}\;f\left(x\right)\;dx$$

Here, *d* (dose) is the exposure concentration over a 1-h immersion challenge, and *I*_*c*_ or *I*_*V*_ is the proportion of individuals infected in either the unvaccinated control group or the vaccine group, respectively. The subscript “hom” denotes a model assuming homogenous susceptibility, where “het” assumes that individuals have susceptibility (*x*) that is distributed according to a beta distribution. *p* is the per-virus particle concentration rate of host infection in microliters per PFU-hour.

We also fitted models that allowed heterogeneity in susceptibility in the unvaccinated control group.

$$I_{C_{\text{het}}}=1-\int_{0}^{\infty }e^{-pd}\;f\left(p\right)\;dp=1-{\left(\frac{1}{1\;+\;\overset{¯}{b}dv}\right)}^{\frac{1}{v}}$$

(2) $$I_{V}=1-\int_{0}^{\infty }\int_{0}^{1}e^{-xpd}\;g\left(x\right)\;f\left(p\right)dx\;dp=1-\int_{0}^{1}{\left(\frac{1}{1\;+\;\overset{¯}{b}xdv}\right)}^{\frac{1}{v}}\;g\left(x\right)\;dx$$

Here, unvaccinated fish have a baseline susceptibility that is distributed according to a gamma distribution. The Laplace transform simplifies the integral in the control (*I*_*c*_) group (19, 72), where *v* is the inverse of the shape parameter and *b* is the mean of the gamma distribution parameterized as shape divided by rate.

For the two-strain challenge experiment, we modified the above equations to reflect differences in the probability of infection with two strains (2), one strain (1), and zero strains (0).

$$I_{C_{2}}={\left(1-e^{-pd}\right)}^{2}$$

$$I_{C_{1}}=2(e^{-pd})\;(1-e^{-pd})$$

$$I_{C_{0}}=e^{-2pd}$$

$$I_{V_{2}}=\int_{0}^{1}{\left(1-e^{-xpd}\right)}^{2}\;f\left(x\right)\;dx$$

$$I_{V_{1}}=\int_{0}^{1}2(e^{-xpd})\;(1-e^{-xpd})\;f\left(x\right)\;dx$$

$$I_{V_{0}}=\int_{0}^{1}(e^{-2xpd})\;f\left(x\right)\;dx$$

Here, there was no support for unvaccinated fish having any variation in susceptibility, and so we assumed a homogeneous susceptibility constant (*p*). Vaccinated fish followed a beta distribution *f* (*x*), determined by shape parameters α and β.

To evaluate the influence of heterogeneity in susceptibility due to vaccination on disease dynamics, we formulated a hypothetical epidemic through a closed population with similar attributes as IHNV using a simple susceptible-infected-recovered transmission model with disease-induced death and where surviving infected individuals recover with sterilizing immunity. We arbitrarily selected values for the transmission rate, recovery rate, and disease-induced mortality rates and varied the transmission rate to explore the influence of *R*_0_ on the size of the outbreak for homogeneous, heterogeneous, and all-or-nothing susceptibility (Fig. S2).

To model this, we assumed that susceptibility lies between 0 and 1. For homogeneous susceptibility, we modeled a vaccinated population where vaccine effectiveness was represented by σ, equal to the mean susceptibility of vaccinated fish from the two-strain experiment.

$$\frac{dV}{dt}=-\text{σ}vVI$$

$$\frac{dI}{dt}=\text{σ}\nu VI-\text{δ}I-\text{γ}I$$

$$\frac{dR}{dt}=\text{γ}I$$

Here, ν is the per capita rate of infection, δ is disease-induced mortality, and γ is the rate at which infected individuals recover from infection. *V*, *I*, and *R* represent the population sizes of vaccinated, infected, and recovered individuals, respectively. For initial conditions, we arbitrarily set the number of vaccinated individuals to 100 and assumed a single infected individual.

To model continuous heterogeneous susceptibility using susceptibility estimates from our experiments, we discretized susceptibility into 300 bins and used the midpoint *x*_*i*_ of the bin to represent the susceptibility of the hosts. By integrating over the lower and upper bounds of each bin, we determined the frequency of individuals in each susceptibility bin or group.

$$\frac{dV(x)}{dt}=-xvV\left(x\right)\int I\left(u\right)\;du$$

$$\frac{dI(x)}{dt}=xvV\left(x\right)\int I\left(u\right)du-\text{δ}I\left(x\right)-\text{γ}I\left(x\right)$$

$$\frac{dR(x)}{dt}=\text{γ}I(x)$$

Here, *x* is susceptibility distributed according to a beta distribution with parameters α = 0.27 and β = 0.49, and each moment of exposure is *x* times more or less likely to infect a fish of susceptibility equal to 1 because each particle to which it is exposed is *x* times as infectious.

To model all-or-nothing susceptibility, the vaccine group was composed of two discrete susceptibility states representing an all-or-nothing mode of vaccine action, *V*_1_ (individuals with no vaccine protection, σ_1_ = 1) and *V*_2_ (individuals with complete vaccine protection, σ_2_ = 0). The number of individuals in each vaccine group was determined from the estimate of mean susceptibility from the two-strain experiment.

$$\frac{dV_{1}}{dt}=-{\text{σ}}_{1}vV_{1}I$$

$$\frac{dV_{2}}{dt}=-{\text{σ}}_{2}vV_{2}I$$

$$\frac{dI}{dt}=\left({\text{σ}}_{1}vV_{1}I\;+\;{\text{σ}}_{2}vV_{2}I\right)-\text{δ}I-\text{γ}I$$

$$\frac{dR}{dt}=\text{γ}I$$

All models were simulated in continuous time using package deSolve (73) in RStudio v.0.99.484 (74).
