# Supplementary material for: Vaccine Effects on Heterogeneity in Susceptibility and Implications for Population Health Management
Source: mBio. 2017 Nov 21;8(6):e00796-17. doi: 10.1128/mBio.00796-17 (PMC5698548; doi:10.1128/mBio.00796-17)
Supplement: TABLE S1 [file mbo006173590st1.docx]

Table S1. Dose-response model comparisons of heterogeneous (beta and gamma) and homogeneous models.

| Model | Mean | Variance | Deviance |
| --- | --- | --- | --- |
| Beta Controls | 0.48622827 | 0.05 | 0.5162943 |
| Beta Vaccine | 0.22 | 0.11 | 2.446055 |
| Gamma Controls | 0.94 | 0.62 | 0.51 |
| Gamma Vaccine | 0.291938 | 7.616156 | 2.446054 |
| Homogeneous Controls | 0.493221 | 0 | 4.780765 |
| Homogeneous Vaccine | 0.002779431 | 0 | 63.50781 |
