# Supplementary material for: Vaccine Effects on Heterogeneity in Susceptibility and Implications for Population Health Management
Source: mBio. 2017 Nov 21;8(6):e00796-17. doi: 10.1128/mBio.00796-17 (PMC5698548; doi:10.1128/mBio.00796-17)
Supplement: TABLE S2 [file mbo006173590st2.docx]

Table S2. Two-strain experiment model comparisons of heterogeneous (beta and gamma) and homogeneous models.

| Model | Mean | Variance | Likelihood |
| --- | --- | --- | --- |
| Beta Controls | 0.3515378 | 0.04748931 | 4.477471 |
| Beta Vaccine | 0.3588346 | 0.1308535 | 4.450734 |
| Gamma Controls | 0.3600537 | 0.06469608 | 4.477471 |
| Gamma Vaccine | 0.4509296 | 0.4408779 | 4.450734 |
| Homogeneous Controls | 0.3075928 | 0 | 5.733158 |
| Homogeneous Vaccine | 0.2498879 | 0 | 12.86737 |
