# Supplementary figures and images for: Vaccine Effects on Heterogeneity in Susceptibility and Implications for Population Health Management
Source: mBio. 2017 Nov 21;8(6):e00796-17. doi: 10.1128/mBio.00796-17 (PMC5698548; doi:10.1128/mBio.00796-17)

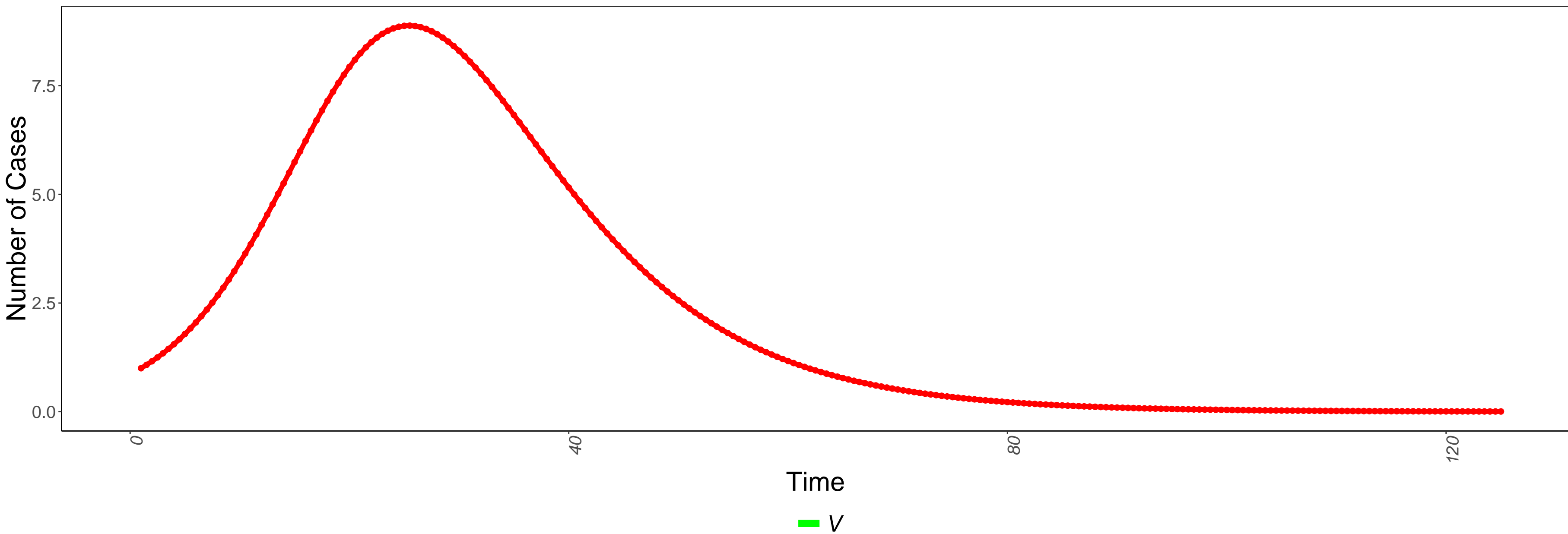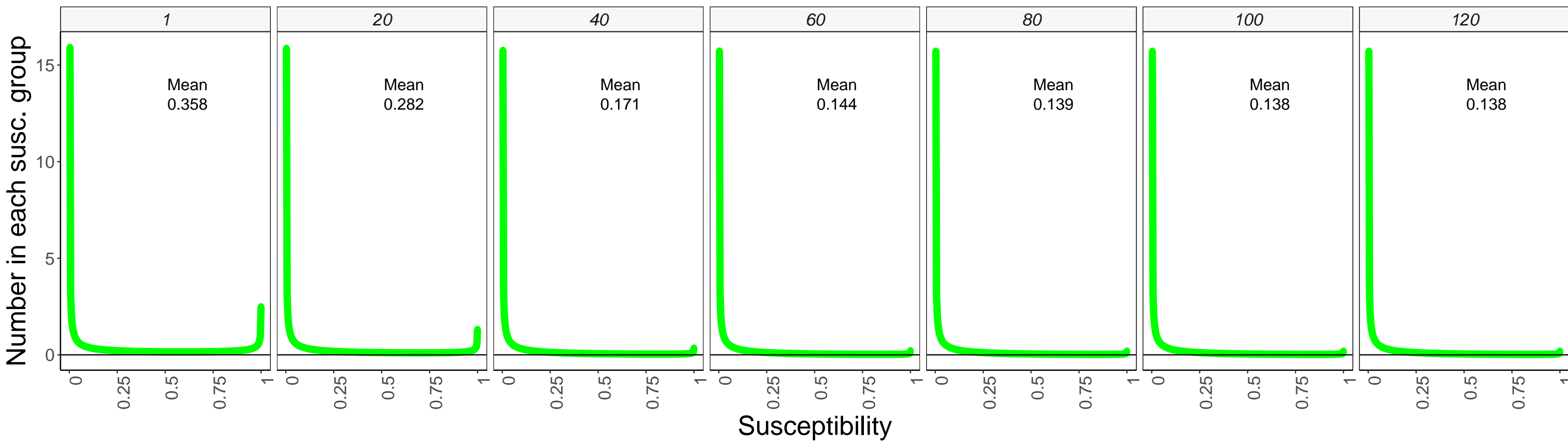

Supplement: FIG S1 [file mbo006173590sf1.pdf]

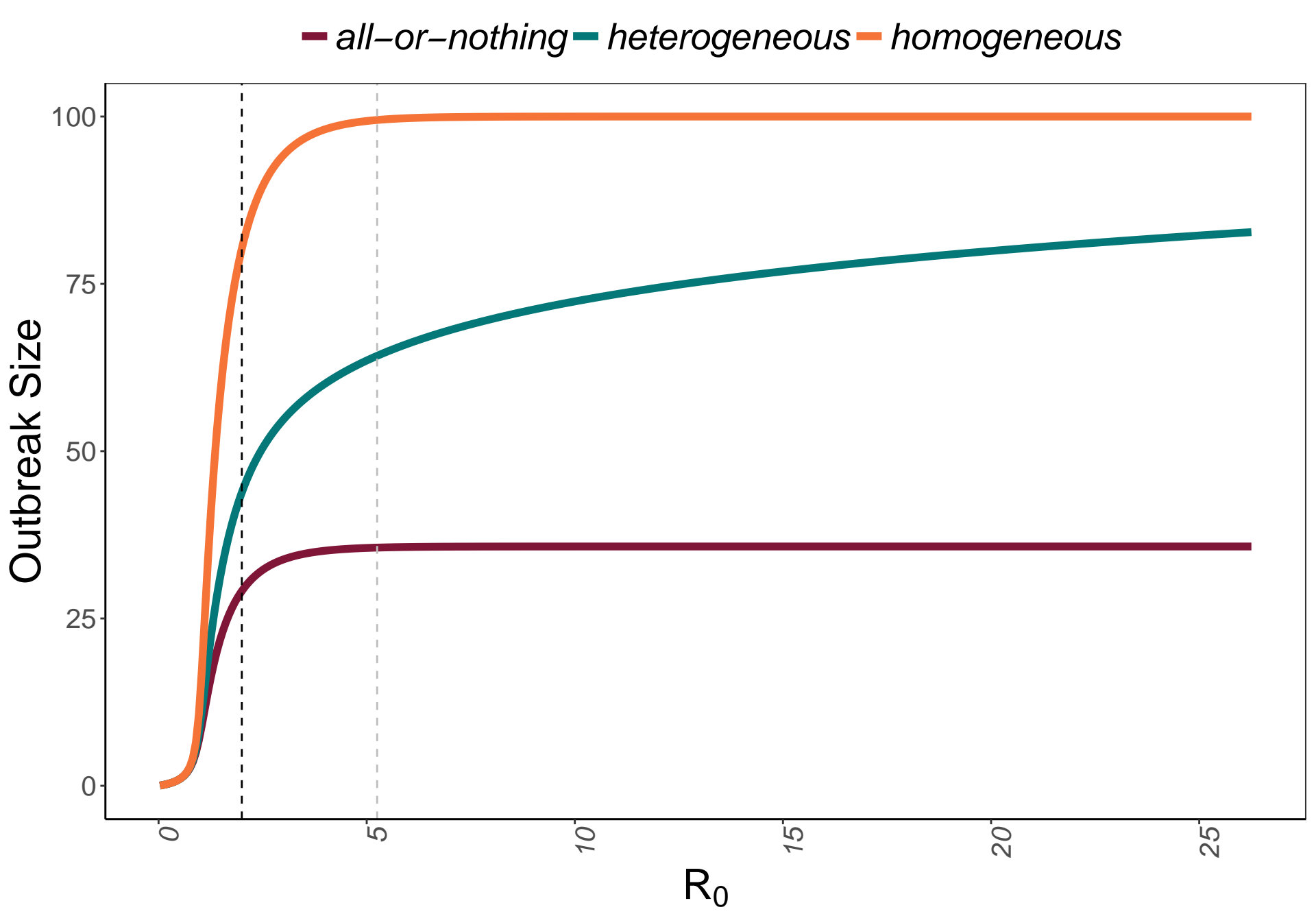

Supplement: FIG S2 [file mbo006173590sf2.pdf]
